# Human cross-tissue cell atlases: unprecedented resources towards systematic understanding of physiology and diseases

**DOI:** 10.1038/s41392-022-01201-w

**Published:** 2022-10-05

**Authors:** Peng Zhang, Shao Li

**Affiliations:** Institute for TCM-X, MOE Key Laboratory of Bioinformatics, Bioinformatics Division, BNRIST, Department of Automation, Tsinghua University, 100084 Beijing, China

**Keywords:** Genome informatics, Systems biology

Recently, three landmark cross-tissue single-cell atlases were published in *Science*, by the Tabula Sapiens Consortium,^1^ Eraslan et al.,^2^ and Domínguez Conde et al.,^3^ respectively, which collectively provided the comprehensive characterization of more than million cells across more than 30 human tissues, along with the deep interpretation of disease-associated genetic variations in the context of tissues and cell types.

Emerging evidences have demonstrated that complex diseases often manifest and involve in diverse cell types across multiple tissues, emphasizing the importance of the cross-tissue single-cell atlases for fully understanding the way in which genetic variation affects disease.^4^ With the development of single-cell RNA-seq (scRNA-seq) technology and foundation of the Human Cell Atlas (HCA) Consortium, massive studies have profiled single-cell landscapes for a broad spectrum of human tissues, revolutionizing our understanding of molecular and cellular mechanism underlying homeostasis and diseased conditions.^5^ However, most of these studies are limited to constructing single-cell atlas for individual organs or diseases, and thus lack the deep investigation of the cellular components and features across tissues and the systematic evaluation of the associations between disease phenotypes and diverse cell types.^4^ Moreover, some of them are also limited by the small size of the atlas, making it difficult to uncover rare cell populations or cellular interactions. Thus, it is urgently needed to construct the large-scale cross-tissue single-cell atlases of the human body to relate cell types or states to the full range of human diseases.

Historically, these three cross-tissue cell atlases, collectively covering more than 500 cell types, were created by researchers from the HCA Consortium with innovative experimental and computational methods. In detail, the cross-tissue atlas created by the Tabula Sapiens Consortium^1^ included scRNA-seq profiles of nearly 500,000 cells from 24 different organs and tissues, through combing Smart-seq and 10x droplet-based sequencing technologies. Meanwhile, the atlas created by Eraslan et al.^2^ included singe-nucleus RNA sequencing (snRNA-seq) profiles of more than 200,000 cells from eight representative organs, where cross-tissue data were integrated by using the conditional variational autoencoder framework. Additionally, given the pivotal role of immune system in tissue homeostasis and disease pathology, the cross-tissue immune cell atlas created by Domínguez Conde et al.,^3^ provided high-quality data for nearly 330,000 immune cells from lymphoid and non-lymphoid organs by using scRNA and paired VDJ sequencing and developing the CellTypist analytic tool, leading to identification of nearly 43 fine-grained cell types and states. These cross-tissue cell atlases motivated efforts to define cellular compartments shared across tissues, characterize the molecular features of broad cell types, and uncover rare and poor-characterized cell types or subsets within specific human tissues, such as interstitial cells of Cajal^2^ and peripheral nervous system cells.^1^ Collectively, these large datasets could help us reveal tissue-shared and tissue-specific cell features, and provide unprecedented insights into cell types or cell states underlying disease pathogenesis.

Firstly, these cross-tissue cell atlases could reveal conserved and variable molecular features and interpret the role of these specializations into organ function, with systematic comparison of cell types across different tissues. Taken fibroblasts as an example, Eraslan et al., demonstrated that fibroblasts across multiple tissues shared common expression program of extracellular matrix constituents, whereas those from lung tissue (lung alveolar fibroblasts) showed distinct features of calcium signaling, indicating their potential migratory or mechanical properties.^2^ Similarly, the Tabula Sapiens Consortium found that endothelial cells from lung, heart, uterus, liver, pancreas, fat, and muscle featured by distinct transcriptional signatures, indicating highly tissue-specialized functions, including the specialized metabolism function for heart endothelial cells and angiogenic function for lung endothelial cell subpopulations.^1^ As for immune cells, Domínguez Conde et al., uncovered that immune cell subsets from different tissues were featured as variable chemokines, reflecting their adaptation to tissue microenvironments.^3^

Secondly, these cross-tissue atlases could also provide insight into associating diseases to specific cell types or states in terms of the pleiotropy and specificity of disease-associated variants.^4^ In this regard, previous scRNA-seq studies from individual organs have associated risk genes with specific cell types by coupling cell atlases and disease-related genetic signals.^5^ Here, Eraslan et al. further systematically related variants to cells and molecular processes by analysis across multiple tissues for both monogenic and complex diseases.^2^ As a typical example of monogenic disorders, the authors focused on muscle diseases and identified finer myocyte subsets linked with different muscle disorders. As for complex diseases, the authors have uncovered associations between disease manifestation and cell types by leveraging the enrichment pattern of disease-related genome-wide association study (GWAS) loci within corresponding cell types across tissues. Interestingly, several less well-documented associations were uncovered, such as adipocytes-atrial fibrillation associations, indicating the potential of these atlases in revealing new disease mechanism. Additionally, the disease-associated cell cluster was uncovered within lung-resident macrophages in the cross-tissue immune cell atlas created by Domínguez Conde et al.^3^

**Fig. 1:**
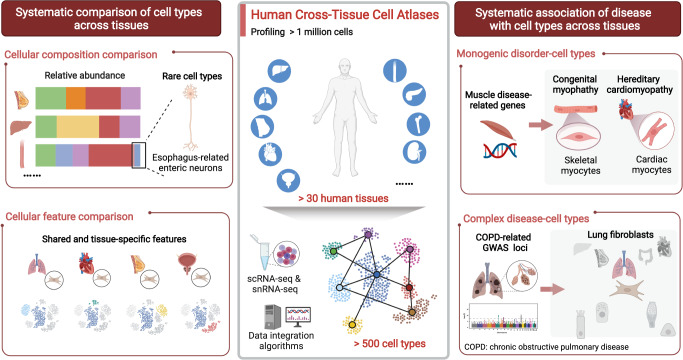
The three large-scale cross-tissue cell atlases, comprising of the scRNA and snRNA-seq profiling of more than 1 million cells from more than 30 human tissues, were constructed by researchers from HCA Consortium. These atlases provide an unprecedented resource for systematic comparison of cell types and systematic association of diseases with cell types across tissues. Herein, the former section mainly included the Cellular composition comparison module that assessed the relative abundances for diverse cell types across tissues and the Cellular features comparison that investigated the tissue-shared and –specific transcriptional features. The latter section mainly contained the Monogenic disorder-cell types module that associating cell types with monogenic disease phenotypes, such as muscle diseases, and the Complex disease-cell types module that associating cell types with complex diseases, such as COPD, gaining deep insights into mechanism underlying human physiology and diseases. The figure is created with BioRender.com

Taken together, these three cross-tissue cell atlases, along with innovative experimental and analytical frameworks, present a significant step toward understanding cross-tissue variation of cellular phenotypes in relation to the function and regulation of disease genes (Fig. 1). Future works should be broadened to include more organs and tissues, and single-cell multi-omics, including single-cell epi-genomics. Furthermore, it would be also needed to develop computational frameworks to integrate multi-layer associations encompassing disease phenotypes, tissues, cell types and genes in a holistic view. Nevertheless, we believed that these seminal works would laid a solid foundation and broadly used molecular reference to explore human biology at single-cell resolution.
